# Medial Frontal White and Gray Matter Contributions to General Intelligence

**DOI:** 10.1371/journal.pone.0112691

**Published:** 2014-12-31

**Authors:** Toshiyuki Ohtani, Paul G. Nestor, Sylvain Bouix, Yukiko Saito, Taiga Hosokawa, Marek Kubicki

**Affiliations:** 1 Psychiatry Neuroimaging Laboratory, Department of Psychiatry and Radiology, Brigham and Women's Hospital, Harvard Medical School, Boston, MA, United States of America; 2 Clinical Neuroscience Division, Laboratory of Neuroscience, Department of Psychiatry, Veterans Affairs (VA) Boston Healthcare System, Harvard Medical School Brockton, MA, United States of America; 3 Department of Psychology, University of Massachusetts, Boston, MA, United States of America; University of Tokyo, Japan

## Abstract

The medial orbitofrontal cortex (mOFC) and rostral anterior cingulate cortex (rACC) are part of a wider neural network that plays an important role in general intelligence and executive function. We used structural brain imaging to quantify magnetic resonance gray matter volume and diffusion tensor white matter integrity of the mOFC-rACC network in 26 healthy participants who also completed neuropsychological tests of intellectual abilities and executive function. Stochastic tractography, the most effective Diffusion Tensor Imaging method for examining white matter connections between adjacent gray matter regions, was employed to assess the integrity of mOFC-rACC pathways. Fractional anisotropy (FA), which reflects the integrity of white matter connections, was calculated. Results indicated that higher intelligence correlated with greater gray matter volumes for both mOFC and rACC, as well as with increased FA for left posterior mOFC-rACC connectivity. Hierarchical regression analyses revealed that DTI-derived FA of left posterior mOFC-rACC uniquely accounted for 29%–34% of the variance in IQ, in comparison to 11%–16% uniquely explained by gray matter volume of the left rACC. Together, left rACC gray matter volume and white matter connectivity between left posterior mOFC and rACC accounted for up to 50% of the variance in general intelligence. This study is to our knowledge the first to examine white matter connectivity between OFC and ACC, two gray matter regions of interests that are very close in physical proximity, and underscores the important independent contributions of variations in rACC gray matter volume and mOFC-rACC white matter connectivity to individual differences in general intelligence.

## Introduction

Healthy cognition has long been assumed to depend on the functional and structural integrity of widely distributed networks of brain regions that allow for efficient and economical coordination and communication of neural signals [1]. In computational neuroscience, neural network models, in their simplest and most basic forms, typically incorporate two key components, individual processing units represented by thresholds of activation and their weighted connections or pathways that are presumed to govern signal transmission [2]. In experimental studies, these basic network components are instantiated on a fine scale to model the encoding of synaptic connections between neurons [3]. At a much broader scale, however, functional and structural elements of neural network models may be investigated by in vivo magnetic resonance imaging (MRI) studies of the brain. Here not only can global brain volume be examined, but, most importantly, regional brain volume of particular neural centers that help to form functional networks can also be quantified.

With conventional MRI, brain volumes of particular regions of interest index structural integrity of gray matter tissue, which includes cell bodies, dendrites and myelinated axons, which from a computational modeling perspective, may be viewed as physical representations of processing units that form specific neural networks. On the other hand, however, with conventional MRI, the integrity of neural network connections is difficult to assess. These neural connections, which have been likened to the structural highways of the brain [4], are physically represented by white matter tissue, defined primarily by the predominance of axonal myelin and supporting glial cells. The difficulty in measurement is that the reconstructed images of white matter architecture appear quite homogeneous [5]. Thus in vivo structural studies now commonly rely on a new technological advancement in MRI, known as diffusion tensor imaging (DTI), which provides for direct measurement and quantification of white matter architecture [6].

DTI reconstructs the white matter pathways of the brain by determining the movement of water molecules in relation to the density and coherence of local brain tissue components, such as cell membrane, axons, and organelles [6], [7]. Most DTI studies implement streamline tractography as a visualization and analytic tool to produce explicit representations of specified brain fiber bundles. These images represent the estimated orientation and direction of fiber tracts derived from the diffusion profile of water in white matter brain tissue. For the majority of white matter, the rate of diffusion is directionally dependent, a property that is referred to as anisotropy. However, there is also isotropic movement of water molecules in the brain (especially in the areas of fiber crossings, gray matter and cerebro-spinal fluid spaces), and streamline tractography will fail to capture direction and orientation of this diffusion. Similarly, streamline tractography does not provide information about the confidence regarding the estimated fiber bundles, and thus uncertainty of the generated tracks caused by increased imaging noise (such as diffusion signal within the gray matter) or complex fiber configurations (such as fiber crossings) are not taken into account. Streamline tractography is thus not an optimal tool for studying white matter connectivity between gray matter regions.

The stochastic tractography method, introduced by Björnemo et al., 2002 [8], is a Bayesian approach that addresses the aforementioned shortcomings of streamline tractography by performing tractography via a probability distribution that accounts for the degree of uncertainty in diffusion tensor fields. This method uses probabilistic models of imaging noise and fiber architecture to infer the underlying fiber configuration, and, because it explicitly models uncertainty, and does not use a pre-determined discrete criterion for generating tracts, the method can effectively generate tracts in regions of low certainty/low anisotropy. Consequently, stochastic tractography can track through fiber crossings and be initiated in gray matter, which makes it a powerful tool to model and measure the anatomy of specific functional networks. In addition, an estimate of the degree of certainty of white matter connection measured along the tract can provide parametric information regarding the strength of anatomical connectivity between two regions of interest (ROI) (as proposed also recently by Kreher et al., 2008 [9]).

In this study, we apply both conventional MRI-derived measures of gray matter and stochastic tractography of white matter using DTI to examine the circuitry of two key brain regions --- orbital frontal cortex (OFC) and anterior cingulate cortex. These two brain regions help to form a neural network that has long been thought to be critical for reinforcement learning and motivation that are so central to healthy neuropsychological functioning [10]–[12]. The OFC is considered among the most polymodal regions of the brain, receiving multi-sensory inputs of taste, smell, auditory, visual, and somatosensory as well as visceral signals, and has direct projections to functionally diverse cortical and subcortical regions, including the amygdala, anterior cingulate cortex, insula, hypothalamus, hippocampus, striatum, as well as its neighboring dorsolateral prefrontal cortex [13]. By virtue of this heterogeneous pattern of connectivity, the orbital frontal lobe is commonly described as a convergence zone for afferents from limbic and heteromodal association regions [14].

Different sub-regions of the orbital frontal cortex show different patterns of connectivity. One important sub-region is the medial orbital frontal cortex (mOFC), with its architecture of Brodmann's area (BA) 13 forming its posterior part, BA 14 forming its medial part, and BA 11 forming its anterior part [15]. Among the strongest connections of the mOFC are those white matter tracts projecting to the anterior cingulate cortex [13]. Originating in midbrain dopamine neurons, these projections furnish both input and output to the dopaminergic rich anterior cingulate cortex and orbital frontal sites [16].

The anterior cingulate cortex, like the orbital cortex, is marked a by a heterogeneous pattern of structural and functional connectivity, which has been divided into cognitive and affective components [17]. The anterior cingulate cortex cognitive division comprises caudal areas 24 and 32, and has been thought to play important roles in working memory and selective attention (e.g., Koski and Pauss, 2000 [18]). In contrast, the rostral section of the anterior cingulate cortex (rACC), located anterior to the genu, includes areas 32, 25 and inferior parts of area 24, and all receive projections from the amygdala that enables coding of the motivational salience of stimuli [17]. However, more recent studies have pointed to limitations in this dorsal-cognitive and rostral-affect dichotomy, with converging evidence of distinct contributions of each of these functional divisions of the anterior cingulate cortex to different forms of emotional processing (see Etkin et al., 2011 [19]).

Hence, both the mOFC and rACC have been thought to play an important role in learning and motivation, which would presumably be integral to the development of general intelligence. Thus, we focused on the general intelligence and hypothesized that the integrity of the structures including mOFC, rACC, and the white matter in the pathway that connect mOFC and rACC can influence general intelligence measured by neuropsychological tests.

Two aims guided the current study. The first aim is to examine within the same subjects both MRI gray matter of the mOFC, rACC and DTI white matter of their direct connections. To address this question, we used stochastic tractography to model white matter connections between the mOFC and the rACC. Methodologically, this study is to our knowledge the first to examine white matter connectivity between OFC and ACC, two gray matter regions of interests that are very close in physical proximity. These DTI-constructed white matter connections are quantified using a scalar measure, referred to as fractional anisotropy (FA), which provides an index of fiber density, axonal diameter, and myelination [20]. Although not as specific to microstructural pathologies as other diffusion indices [21], FA is regarded as the most reliable measure of white matter properties, and growing evidence of its validity is seen in its association with abnormalities of cognition in psychiatric samples [22], [23]. In addition, MRI gray matter volumes of the mOFC and the rACC provide a distinct measure of individual variation in microstructural organization independent of DTI-derived FA values used to assess white matter connectivity. The second aim of the study is to address the external validity of these structural brain imaging measures by examining their relationship to neuropsychological performance, particularly on tests of general intelligence. Haier et al. (2004) [24] reported individual differences in general intelligence, which were correlated strongly with greater volumes in both gray matter and white matter across primarily frontal, temporal, and parietal regions. In addition, FA global white matter integrity in the white matter pathways made contributions to general intelligence [25]. These studies suggested the association between gray and white matter structures and general intelligence.

## Materials and Methods

### Subjects

Twenty-six healthy subjects were recruited through advertisements in local newspapers, and interviewed using the SCID-NP [26]. Exclusion criteria included a history of neurological illness, alcohol/drug dependence in the last 5 years or abuse in the past year, current medications with deleterious effects on neurological or cognitive functions, and first degree relatives with an Axis I disorder. All subjects evinced the ability and desire to cooperate with the procedures, as confirmed by written informed consent. The subjects were all right handed males, the mean age was 38.62±10.61, education (years) was 15.04±1.89, subjects' own socio-economic status (SES) [27] was 1.96±0.68, and parental SES was 2.29±1.20. This study was approved by the VA Boston Healthcare System and Harvard Medical School Institutional Review Boards. Written informed consent was obtained from all subjects prior to study participation.

### Neuropsychological Assessment

The neuropsychological battery consisted of three neuropsychological tests: (1) Wechsler Adult Intelligence Scale-Third Edition (WAIS-III; Wechsler, 1997 [28]); (2) Wisconsin Card Sorting Test (WCST; Heaton, 1981 [29]); and (3) Trail Making Test (TMT; Reitan, 1992 [30]). The WAIS-III yields intelligent quotients for verbal (VIQ), performance (PIQ), and full-scale (FSIQ). The WCST, a well-known test of executive functions of planning, self-monitoring, and response-regulation yields the following dependent measures: (a) the number of categories achieved (0-6), (b) perseverative errors; and (c) non-perseverative errors. The TMT, a timed pencil-and-paper test, yields dependent measures of visual scanning for Trails A and working memory processes of attentional control for Trails B [31]. Most of the clinical and neuropsychological evaluations were performed at protocol entrance, with MRI scans occurring one week later.

### MRI Protocol

DTI data was collected using a 3Tesla GE Echospeed system (General Electric Medical Systems, Milwaukee, WI). Scans were acquired with echo planar imaging (EPI) DTI Tensor sequence, and a double echo option to reduce eddy-current related distortions [32], [33]. An 8 Channel coil and ASSETT (Array Spatial Sensitivity Encoding techniques, GE) with a SENSE-factor (speed-up) of 2 was used to reduce the impact of EPI spatial distortion. In order to accommodate for higher spatial resolution required by this study, the product GE sequence has been modified. We acquired 85 axial slices parallel to the AC-PC line covering the whole brain in 51 diffusion directions with b = 900, and 8 baseline scans with b = 0. Scan parameters were as follows: TR 17000 ms, TE 78 ms, FOV 24 cm, 144×144 encoding steps, 1.7 mm slice thickness, producing isotropic 1.7×1.7×1.7 mm voxels. In addition to DTI scans, a structural MRI acquisition protocol that includes two MRI pulse sequences was also used. The first results in contiguous spoiled gradient-recalled acquisition (fast SPGR) with the following parameters: TR = 7.4 ms, TE = 3 ms, TI = 600, 10degree flip angle, 25.6 cm 2field of view, matrix = 256×256; the voxel dimensions are 1×1×1 mm. The second- XETA (e Xtended Echo Train Acquisition) produces a series of contiguous T2-weighted images (TR = 2500 ms, TE = 80 ms, 25.6 cm 2field of view). Voxel dimensions are also 1×1×1 mm. This latter sequence is used as the additional channel of information for brain segmentation.

### Image Processing

#### ROI creation

Freesurfer (http://surfer.nmr.mgh.harvard.edu), a semiautomatic segmentation method, was used to extract bilateral rACC and bilateral mOFC regions. Then, FNIRT - FMRIB's Non-linear Image Registration Tool (http://www.fmrib.ox.ac.uk/fsl/fnirt) was used to co-register anatomical scans along with corresponding ROIs to DTI space. Additionally, the mOFC was divided into anterior (ant mOFC) and posterior (post mOFC) regions [34]. The anterior border of the post mOFC was defined as the most anterior coronal slice that contained rACC.

#### Stochastic Tractography Generation

**Figure 1:**
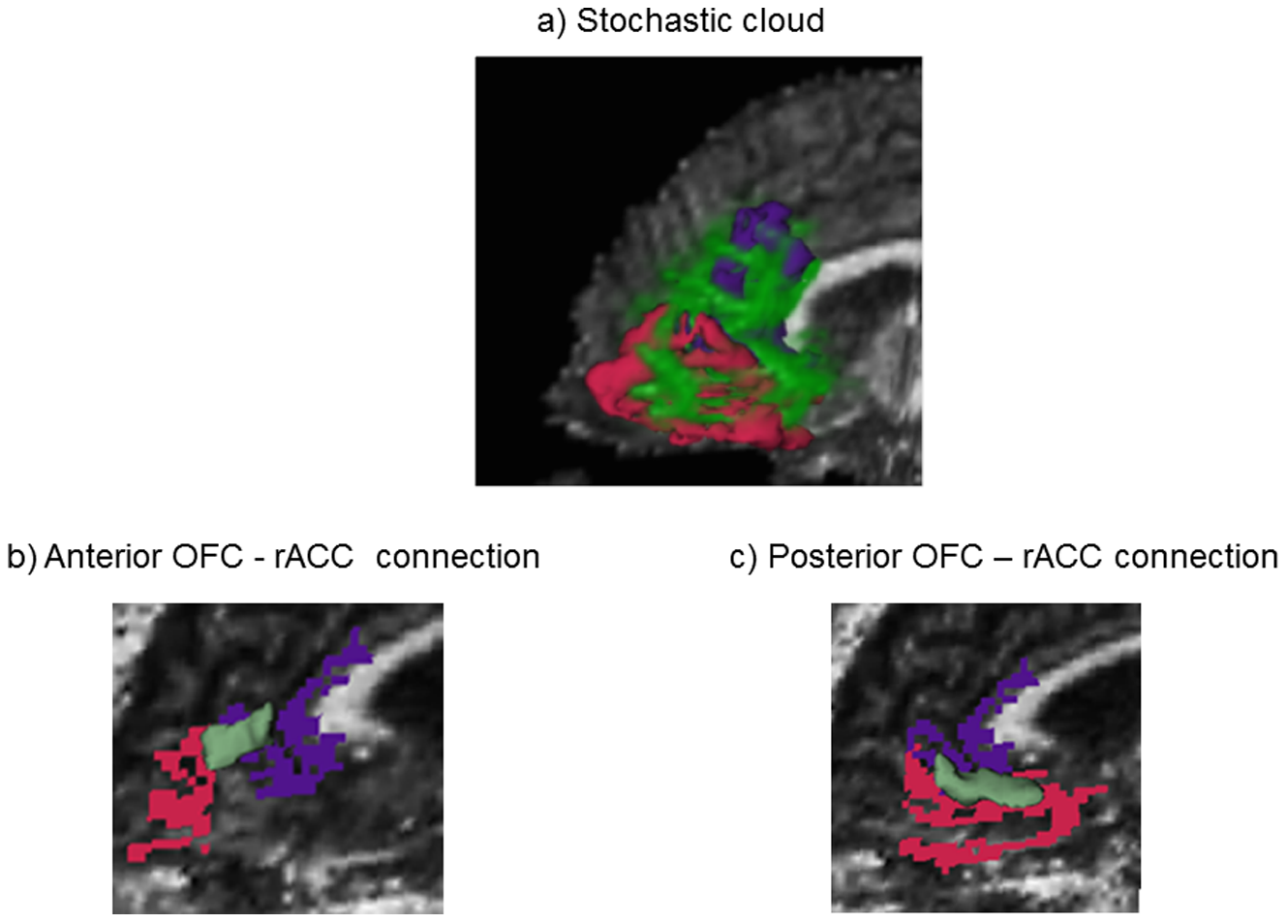
The stochastic cloud connecting the medial orbitofrontal cortex (mOFC) and the rostral anterior cingulate cortex (rACC). The red structure is the mOFC, the purple is the rACC, and the light green is the stochastic cloud connecting the mOFC and rACC.

After the ROIs (i.e. bilateral ant mOFC, post mOFC, and rACC) were transferred to DTI space, stochastic tractography (part of Slicer3 software (www.slicer.org)) was performed to generate WM connections. For total connectivity measures, tracts were seeded first in the mOFC region and filtered through the rACC, then seeded in the rACC and filtered through the mOFC region, and both connections were used for the subsequent analyses. This procedure was performed first for the ant mOFC-rACC connection and then the post mOFC-rACC connection. 500 seeds were placed per voxel for stochastic tractography generation. For establishing reliability of seeding, see Kubicki et al. (2011) [5]. No stopping criterion for tractography was used in this study. We obtained a map that showed the probability of connection, based on the number of tracts per voxel divided by the total number of tracts generated. In order to eliminate unlikely paths, the probability map was assigned a 10% threshold [5], [35]. Mean FA, was calculated within each tract mask to determine the properties of the WM connecting the mOFC and rACC. The ROIs and stochastic cloud are displayed in Fig. 1.

### Data Analysis

ROI volumes, as well as measures of WM connectivity between these ROIs, were extracted and subjected to quantitative analysis. Data were analyzed using the Statistical Package for Social Sciences (SPSS v.20.0). Mean FA in the four connections was analyzed and, in order to examine the effect of volume on the FA values, correlations between the ROI volumes and the mean FA values of the tracts generated from the ROIs were examined by Pearson's correlation coefficient. We also examined the association between ROI volumes and neuropsychological test scores. In this correlation analysis, we used the Intracranial Content (ICC) to calculate relative volumes [(absolute volume/ICC × 100] and used relative volumes instead of absolute volumes in order to control for individual head size. Then, we examined the association between mean FA values for each of the four connections and neuropsychological test scores using Pearson's correlation coefficient. Lastly, we used hierarchical regression to further examine the significant univariate relationships of full-scale intelligence with the FA in the connection that showed a significant association with the Full Scale IQ, and with the gray matter volume of the ROI that showed a significant association with the Full Scale IQ. Since the purpose of this study was to investigate the relationship between white and/or gray matter structures and the general intelligence, we have limited the hierarchical regression analysis only to the Full Scale IQ. We used p<0.05 as the cutoff for reporting statistical significance, as we considered these analyses exploratory, however, within the constraints of the anatomical assumptions regarding medial frontal network.

## Results

Table 1 presents mean FA values for mOFC-rACC white matter connectivity. FA values of individual connections were submitted to a 2×2 repeated-measures ANOVA with factors of side (left/right), and coronal plane (anterior/posterior). FA values were significantly greater for posterior than anterior connections, F (1, 25) = 5.68, p = 0.025, Partial Eta Squared  = 0.185, especially for right posterior mOFC-ACC connections, as reflected in the statistically significant interaction of side×coronal plane, F (1, 25) = 5.71, p = 0.025, Partial Eta Squared  = 0.186.

**Table 1:** White matter integrity and gray matter volume.

	Left		Right	
Fractional Anisotropy a)	Mean	SD	Mean	SD
Anterior mOFC – rACC	0.413	0.059	0.387	0.065
Posterior mOFC – rACC	0.425	0.063	0.435	0.061
Gray matter volumes b)	Mean	SD	Mean	SD
Anterior mOFC	1.294	0.479	1.820	0.642
Posterior mOFC	3.658	0.702	3.502	0.495
rACC	2.335	0.546	2.019	0.347

Abbreviations: OFC, orbitofrontal cortex; ACC, anterior cingulate cortex, mOFC, medial orbitofrontal cortex; rACC, rostral part of anterior cingulate cortex.

a) Mean fractional anisotropy values for bilateral mOFC-rACC white matter connections.

b) Gray matter volumes for the ROIs (i.e. anterior mOFC, posterior mOFC, and rACC).

Gray matter volumes for anterior and posterior mOFC are included in Table 1. ANOVA indicated largest gray matter volumes for left posterior mOFC, as reflected in the statistically significant interaction of side×coronal plane, F (1, 25) = 9.20, p = 0.006. Left rACC gray matter volume was also significantly greater than right rACC gray matter, t (25) = 2.733 p = 0.011.

Mean FA values for mOFC-rACC connectivity did not correlate with any of the MRI volumes. In addition, age did not correlate with any gray or white matter measures. These data suggested that white matter properties of mOFC-rACC may be independent of both age and mOFC and rACC gray matter volumes.

**Figure 2:**
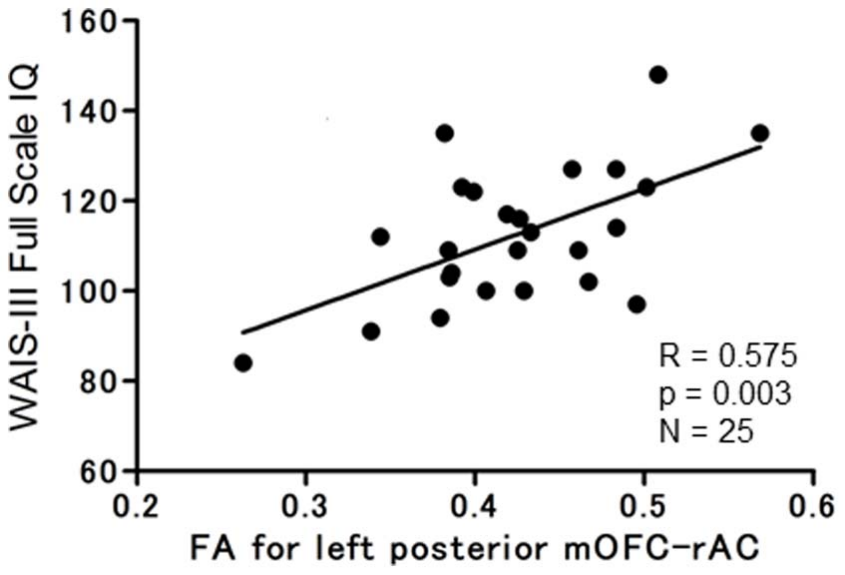
Scatter plot of correlations between WAIS-III Full Scale IQ and left posterior mOFC-rACC FA values (N = 25).

Table 2 presents neuropsychological test scores and Table 3 presents the correlation matrix between the WAIS-III Full Scale IQ and the white matter and gray matter measures for the healthy participants. For DTI, increased FA for left posterior mOFC-rACC correlated significantly with higher scores for WAIS-III Full Scale IQ (r = 0.575, p = 0.003), WAIS-III Verbal IQ (r = 0.504, p = 0.039), and WAIS-III Performance IQ (r = 0.597, p = 0.015). Fig. 2 presents a scatter plot of WAIS-III Full Scale IQ and left posterior mOFC-rACC FA values for 25 participants. For MRI, increased right anterior mOFC gray matter volume correlated with higher WAIS-III Performance IQ (r = 0.526, p = 0.036), as did increased left rACC gray matter volume correlate significantly with higher WAIS-III Verbal IQ (r = 0.573 p = 0.016) and WAIS-III Full Scale IQ (r = 0.394, p = 0.05). Performance on the other neuropsychological tests did not correlate with the structural brain imaging measures.

**Table 2:** Neuropsychological test scores.

Neuropsychological tests	Mean	SD
WAIS-III		
Full Scale IQ	112.6	15.2
IQ for verbal	113.8	16.1
IQ for performance	108.3	19.5
WCST		
Number of category achieved	5.5	1.3
Perseverative errors	11.5	9.8
Non-perseverative errors	10.4	10.4
TMT		
Trail A time spent	27.0	5.5
Trail A errors made	0.1	0.3
Trail B time spent	63.8	27.6
Trail B errors made	0.2	0.5

Abbreviations: WAIS-III, Wechsler Adult Intelligence Scale-Third Edition (WAIS-III; Wechsler, 1997); IQ, intelligent quotient; WCST, Wisconsin Card Sorting Test (WCST; Heaton, 1981); TMT, Trail Making Test (TMT; Reitan, 1992).

**Table 3:** Correlations between the ROI volumes or mOFC-rACC FA and WIAS-III full scale IQ.

	Left		Right	
	Pearson's R	P value	Pearson's R	P value
Volumes				
Anterior mOFC	−0.189	0.366	0.142	0.499
Posterior mOFC	0.213	0.306	0.048	0.821
rACC	0.394	0.05	0.048	0.821
Mean FA				
Anterior mOFC-rACC	0.054	0.798	−0.354	0.083
Posterior mOFC-rACC	0.575	0.003	0.224	0.283

Abbreviations: ROI, region of interest; WAIS-III, Wechsler Adult Intelligence Scale-Third Edition (WAIS-III; Wechsler, 1997); IQ, intelligent quotient; FA, fractional anisotropy; mOFC, medial orbitofrontal cortex; rACC, rostral anterior cingulate cortex

When both of left posterior mOFC-ACC FA and left rACC gray matter imaging measures are entered as predictors in a hierarchical multiple regression model, left posterior mOFC-ACC FA (Beta = 0.538, t = 3.57, p = 0.003) and left rACC gray matter (Beta = 0.334, t = 2.09, p = 0.049) each accounted for a significant portion of variance in WAIS-III Full-Scale IQ scores. For the WAIS-III Full-Scale IQ, left posterior mOFC-ACC FA produced a significant R square change of.331 (F = 11.38, df = 1, 23, p = 0.003) as did left rACC gray matter (F = 4.35, df = 1, 22, p = 0.049). WAIS-III Full-Scale IQ and left posterior mOFC-ACC FA revealed a partial correlation value of 0.582 and a semi-partial correlation value of 0.535. These values indicated that left posterior mOFC-ACC FA uniquely accounted for 34% and 29% of the variance in WAIS-III Full-Scale IQ scores. For WAIS-III Full- Scale IQ and left rACC gray matter, hierarchical regression revealed a partial correlation value of 0.406 and a semi-partial correlation of 0.332. These values indicated that left rACC gray matter uniquely accounted for 16% and 11% of the variance in WAIS-III Full Scale IQ scores.

## Discussion

The current study found that each brain structural element --- gray matter volume and white matter integrity --- was independently associated with general intelligence, and together the mOFC-rACC circuitry accounted for 30% of variance of general intelligence. The mOFC and rACC represent two key brain regions that help form a widely-distributed neural network that has long been thought to be critical for reinforcement learning and motivation that are so central to healthy neuropsychological functioning [10]–[12]. Thus, the study addressed the structural integrity of mOFC-rACC circuitry as measured by both MRI gray matter volume of each of these regions and DTI FA of their white matter connections.

First, MRI-derived gray matter volumes for the mOFC and rACC did not correlate with DTI FA measures of white matter integrity of their connecting pathways. The structural imaging data thus strongly suggest that MRI gray matter volume and FA white matter integrity each provide independent measures of distinct aspects of mOFC-rACC circuitry. From a computational modeling perspective, mOFC and rACC may each represent key processing centers or hubs of a larger extended network that plays an important role in learning and motivation. Further, the data showed structural differences within these brain regions, favoring a left-sided advantage in gray matter volumes for both mOFC and rACC. In addition, for OFC subdivisions, left posterior mOFC showed the largest gray matter volumes. Thus the MRI measures in this study were sensitive to significant anatomical variation within both mOFC and rACC. Such variation may serve as important structural constraints in the development of realistic neural network models, perhaps in formulating algorithmic or computational power of constituent processing units [3].

The current study also implemented stochastic tractography, the most effective diffusion tensor imaging method, in order to investigate white matter connections, bilaterally, between these adjacent gray matter regions. Here FA, which reflects the integrity of these white matter connections, also showed significant anatomical variation, with FA values greater for posterior than anterior connections, particularly for right-sided pathways. These, too, may provide a unique source of information regarding neural circuitry of mOFC and rACC, particularly in relation to the strength of the white matter pathways connecting these two adjacent gray matter regions.

It remains to be determined as to how these anatomical variations in white matter architecture that link these neighboring regions influence the dynamics and function of the wider network. However, the degree of local correlated activity may vary as a function of the strength of the neural circuitry connecting mOFC and rACC regions, and these structural elements likely influence the transmission of coordinated signals across the wider network [3]. Thus, taken together, these brain imaging measures are highly sensitive to variation in white matter and gray matter of the mOFC and rACC. And for neural modeling, these anatomical features may pose important structural constraints for simulating the dynamics and function of local neural networks and the system as a whole.

The current study also examined the relationship of these structural brain imaging measures to neuropsychological performance. The strongest empirical relationship was observed for neuropsychological measures of intellectual abilities with both FA and gray matter volumes of the mOFC and rACC circuitry. For example, for DTI, increased FA for left posterior mOFC-rACC correlated significantly with higher scores for measures of full-scale, verbal and performance intellectual abilities. For MRI, increased right anterior mOFC gray matter volume correlated with higher performance intelligence, as did increased left rACC gray matter volume correlate significantly with measures of verbal and full-scale intelligence.

Follow-up of these general univariate relationships by multiple regression analyses allowed for the direct comparison of the relative contributions of white mater and gray matter to overall, full-scale intelligence. The results indicate that although both white matter and gray matter each make a statistically significant and specific contribution to intelligence, DTI-derived FA of left posterior mOFC-rACC uniquely accounted for 29% to 34% of the variance in IQ, in comparison to 11% to 16% uniquely explained by MRI volume of the left rACC. Thus, these results indicated that white matter connectivity linking left posterior mOFC and rACC, along with left rACC gray matter volume, together, accounted for up to 50% of the variance in overall intelligence. As such, left posterior mOFC-rACC white matter connectivity accounted for twice as much of the variance in IQ than did left rACC gray matter volume.

These findings offer key methodological and theoretical implications. First, from a methodological perspective, the data provide evidence of the validity for both neuropsychological measures of intelligence and brain structural imaging measures of white matter integrity and gray matter volume. Indeed, that the findings demonstrate higher levels of intelligence correspond with increases in both white matter connectivity of the mOFC-rACC, as well as mOFC and rACC gray matter volume underscore the validity of the multimodal approach used in this study of combining brain structural imaging and neuropsychology. In addition, the current findings may also be viewed as lending particular evidence for the validity of the stochastic tractography method implemented in this study to generate white matter connections as measured by FA. In fact, FA of the left posterior mOFC-rACC, derived from stochastic tractography, proved to be the strongest predictor of neuropsychological performance, uniquely accounting for 34% of the variance in intelligence. In contrast, individual differences on neuropsychological tests of executive function did not correlate with the structural brain imaging measures.

Methodologically, the current study is to our knowledge the first to examine white matter connectivity between OFC and ACC, two gray matter regions of interests that are very close in physical proximity. Indeed, reliable measures of white matter connectivity are extremely difficult for regions of interest such as the OFC and ACC that are so structurally situated. Here, the degree to which the diffusion of water molecules is directionally restricted by white matter architecture is difficult to reconstruct and quantify with most DTI methods. The current study resolved this methodological problem by using stochastic tractography, as this relatively new visualization technique incorporates probabilistic models of imaging noise and fiber architecture to infer the underlying fiber configuration. That is, stochastic tractography explicitly models uncertainty, and does not use a pre-determined discrete criterion for generating tracts. The current results showed that stochastic tractography effectively generated tracts in regions of high uncertainty/low anisotropy, such as the mOFC-rACC pathways.

Second, an important theoretical question in human neuropsychology has long centered on the extent to which individual differences in brain structure and function map onto individual differences in cognitive performance. The probative findings of the current study demonstrated a direct linear relationship between individual differences in IQ with individual differences in two distinct, independent measures of brain structure, posterior mOFC-rACC white matter connectivity and left rACC gray matter volume. These findings are most consistent with prior MRI studies of general intelligence that have typically not included DTI-derived FA values of white matter. For example, in an early MRI twin study, Thompson et al. (2001) [36] reported gray matter volume of the frontal lobe that was linked to intelligence and to be under strong genetic control (see also Gray and Thompson, 2004 [1]). Of further note, Jung and Haier (2007) [37] proposed a neural structural theory of intelligence that posits that greater gray and white matter volumes in widespread parietal-frontal brain networks correspond to higher levels of WAIS full-scale IQ. In a Voxel-based Morphometry (VBM) MRI study of gray matter volumes in 405 undergraduate students, Colom et al. (2009) [38] tested the Jung and Haier (2007) model [37], known as the parieto-frontal integration theory of intelligence (P-FIT). Their results provide empirical support for the P-FIT, as scores on measures of general intelligence correlated with increased gray matter volumes in multiple brain areas, especially dorsolateral prefrontal (Brodmann areas 9-10 and 46) cortex, Broca's and Wernicke's areas, parietal somato-sensory cortex, and visual association cortex. In addition, as Colom and colleagues emphasized, the P-FIT ascribes a key role to the arcuate fasciculus, a prominent white matter tract connecting posterior area of the temporal-parietal junction with the frontal lobe. However, as Colom also pointed out, VBM used in their study may not be the best technique to test the P-FIT prediction of white matter variations in the arcuate fasciculus as central to individual differences in intelligence.

Other imaging studies have emphasized the role of both white and gray matter as the infrastructure for efficient neuronal transmission in support of higher intelligence [39]–[41]. Studies have also reported positive correlations of general intelligence and white matter integrity calculated by FA derived from DTI [42], [43]. For example, in a healthy pediatric cohort, Schmithorst et al. (2005) [44] examined the relationship of FA across the cortical lobes with scores on the Wechsler in 47 children between the ages of 5 and 18. Their results showed a significant correlation between higher IQ scores and FA, bilaterally, within frontal and occipital-parietal regions, an expanse of the cortex for which these researchers proposed comprised the white matter tracts of the arcuate fasiculus. Based on these findings, Schmithorst et al. concluded that the integrity of white matter pathways of the arcuate fasciculus linking Broca's area and Wernicke's area may be especially sensitive to individual differences in intelligence in their pediatric sample.

More recently, in a sample of 420 older adults (228 men, 192 women) between the ages of 71 and 73, Penke et al. (2012) [25] used quantitative tractography to segment 12 major white matter pathways and calculated their corresponding FA values. They then submitted these FA values to a principal component analysis from which they extracted a factor-analytically derived measure of global white matter tract integrity of the brain. They applied structural equation modeling (SEM) to examine the relationships among latent factors of white matter tract integrity and latent factors of general intelligence and information-processing speed as measured by six WAIS subtests. Their results indicated that FA global white matter integrity and two additional white matter integrity biomarkers each made independent contributions to general intelligence, and together accounted for 10% of the variance in general intelligence. In addition, their SEM findings showed that this relationship of white matter tract integrity and general intelligence was completely mediated by information processing speed.

The current findings thus expands upon these studies by examining, within the same subjects, the relationship between general intelligence and both regional gray matter volumes of the rACC and the mOFC, as well as white matter integrity of their connecting pathways. Furthermore, the current results support our hypothesis that the integrity of the structures including mOFC, rACC, and the white matter connecting mOFC and rACC can influence general intelligence measured by neuropsychological tests. Both the mOFC and rACC have been thought to play important roles in learning and motivation. More specifically, posterior mOFC contributes to motivational processes [45], while the anterior granular OFC subregions showed a greater association with cognitive tasks [46]. In addition, rACC is primarily involved in assessing the salience of motivational and emotional information and regulating emotional responses [47], [48], and the OFC and ACC are thought to interact together to form a network involved in emotional processing [49] (e.g., de Marco et al., 2006). Thus, based on those findings and our results, we speculate that the integrity of the posterior mOFC-ACC FA and rACC gray matter volume impact general intelligence through the motivation and emotional processing. Although, the precise functional role mOFC-rACC circuitry plays within the widely distributed, broader neural networks of intelligence remains to be empirically tested, the observed relation of intelligence with increased FA of the left posterior mOFC-rACC pathways may reflect highly-structured, tightly-aligned axonal tracts that promote efficient transmission of electrical signals between these neighboring brain regions. Such effective neural communication might in turn be a key element in general intelligence. Individual variations in the strength of these connections as measured by FA may map onto individual variations in general intelligence. So too might individual variations in gray matter correspond to individual variations in general intelligence, as this could suggest that higher intelligence is supported by greater neuronal density of the rACC and mOFC processing units.

These theoretical interpretations of the current findings, however, wait future empirical testing. And while consistent with previous research, any theoretical interpretations are limited by the small sample size of the current study. An additional constraint of the current study is the failure of the structural brain imaging measures to correlate with neuropsychological tests scores on measures of executive function, which are both anatomically and cognitively related to general intelligence. Also important to bear in mind is that the present study focused on two discrete brain regions, examining correlations of their gray matter volume and the integrity of their white matter connections with neuropsychological performance. Therefore, the possibility of the type I error in the multiple correlation analysis could not be dismissed. Moreover, many other factors including environment, might also have effect on intelligence. Thus, although these univariate correlations were followed-up by statistically rigorous tests of hierarchical regressions that allowed for the partitioning of general intelligence into specific and unique sources of variance derived from structural brain imaging, future studies with larger samples are needed to demonstrate the reliability and validity of these findings.

## Conclusions

This study examined WM connectivity between OFC and ACC. These two gray matter regions of interests are part of a wider neural network, which plays an important role in general intelligence and executive function. Methodologically, this study is to our knowledge the first to examine WM properties of those connections. The current results underscore the important independent contributions of variations in rACC gray matter volume and mOFC-rACC white matter connectivity to individual differences in general intelligence. Further studies with larger samples are expected to confirm the reliability and validity of the present results.
